# Development and Preliminary Validation of a Tool to Assess Barriers and Facilitators to Participation in Adjuvant Endocrine Therapy Switch Trials

**DOI:** 10.3390/curroncol33060361

**Published:** 2026-06-16

**Authors:** Silvia Belloni, Paola Tiberio, Emanuela Mencaglia, Alice Silvia Brera, Chiara Giacon, Chiara Benvenuti, Flavia Jacobs, Mariangela Gaudio, Rosalba Torrisi, Armando Santoro, Rosario Caruso, Cristina Arrigoni, Alberto Zambelli, Rita De Sanctis

**Affiliations:** 1 Department of Public Health, Experimental and Forensic Medicine, Section of Hygiene, University of Pavia, 27100 Pavia, Italy; cristina.arrigoni@unipv.it; 2 Medical Oncology and Hematology Unit, IRCCS Humanitas Research Hospital, 20089 Rozzano, Italy; paola.tiberio@cancercenter.humanitas.it (P.T.); emanuela.mencaglia@gmail.com (E.M.); chiara.benvenuti@asst-pg23.it (C.B.); flavia.jacobs@istitutotumori.mi.it (F.J.); mariangela.gaudio@cancercenter.humanitas.it (M.G.); rosalba.torrisi@cancercenter.humanitas.it (R.T.); armando.santoro@cancercenter.humanitas.it (A.S.); 3 Fondazione IRCCS Policlinico San Matteo, 27100 Pavia, Italy; a.brera@smatteo.pv.it (A.S.B.); c.giacon@smatteo.pv.it (C.G.); 4 Department of Medical Oncology, ASST Papa Giovanni XXIII, 24127 Bergamo, Italy; azambelli@asst-pg23.it (A.Z.); rdesanctis@asst-pg23.it (R.D.S.); 5 Department of Biomedical Sciences, Humanitas University, 20072 Pieve Emanuele, Italy; 6 Department of Biomedical Sciences for Health, University of Milan, 20133 Milan, Italy; rosario.caruso@unimi.it; 7 Health Professions Research and Evidence Transfer Unit, IRCCS MultiMedica, 20099 Sesto San Giovanni, Italy

**Keywords:** breast cancer, clinical trial, adjuvant endocrine therapy switching, decision making, scale, validity

## Abstract

Improving accrual to clinical trials is critical in oncology, particularly in studies evaluating treatment de-escalation or modification strategies, such as endocrine therapy (ET) switch trials in breast cancer. In disease-free patients who have completed an initial period of standard adjuvant ET, decision-making is shaped by individual, clinical, and contextual factors that may act as barriers and facilitators to trial participation. The lack of validated instruments complicates the comprehensive evaluation of these determinants. Therefore, our study aimed to develop and validate a scale for measuring barriers and facilitators to clinical trial participation in adjuvant endocrine therapy switch trials (CAMBRIA-1 and EMBER-4) among breast cancer patients. Therefore, a multiphase study was undertaken: phase one focused on item generation through literature review and expert consensus; phase two assessed preliminary validity through content and face validation (S-CVI and CVR) and pre-testing (Cronbach’s alpha). The final scale encompassed 17 items and exhibited strong evidence of face and content validity (S-CVI = 0.78 and CVR > 0.725 for all items) and internal consistency (Cronbach’s alpha = 0.874). Since preliminary internal consistency has been established, this tool may support future psychometric refinement and large-scale investigations. An understanding of these determinants may inform trial enrolment and drug development in breast cancer management, as well as tailor patient engagement strategies.

## Simple Summary

Clinical trials have greatly advanced novel findings in breast cancer research. Nevertheless, including patients in randomised controlled trials remains critical, especially when a treatment is proposed as a further option. Decision-making in breast cancer treatment often involves balancing several personal, medical, and contextual factors (i.e., barriers and facilitators) that serve as determinants and influence health outcomes. However, assessing barriers and facilitators to clinical trial participation is currently difficult due to the lack of valid and reliable tools. This study provides a development and initial validation of a scale measuring barriers and facilitators to participation in adjuvant endocrine therapy switch trials (CAMBRIA-1 and EMBER-4) among patients with breast cancer. The final version of our developed scale encompassed 17 items and exhibited evidence of face and content validity (S-CVI = 0.78 and CVR > 0.725 for all items) and internal consistency (Cronbach’s alpha = 0.874). This tool may support future large-scale psychometric evaluations.

## 1. Introduction

Breast cancer (BC) is the most frequently diagnosed cancer in women and the second leading cause of death in women worldwide [1]. Despite its high incidence, BC has a comparatively favourable prognosis, thanks to continuous advances in systemic and local therapies that have significantly improved survival [2], with standards of care evolving rapidly as new agents are introduced. In this scenario, clinical trials play a pivotal role in further improving BC prognosis and outcomes, moving towards more efficient targeted strategies (i.e., umbrella, basket, and platform trials) [3,4,5,6,7,8]. Enrolment in clinical trials was associated with improved quality of life and clinical outcomes up to 4 years post-diagnosis among BC patients [9].

Clinical trial participation remains a critical issue in oncology, with poor accrual as the most common reason for early trial termination, accounting for 34.5% of terminated trials [10,11]. A recent study of BC patients indicated a 52% eligibility rate and only a 63% participation rate among those eligible [12]. Along the participation cascade, multiple junctures can influence the trial enrolment process, including patient eligibility and structural, contextual, and clinician-related factors [12,13,14,15]. Within this framework, patients’ willingness to participate is a key determinant of recruitment [16,17]. Willingness reflects a complex interplay of influences commonly described as barriers and facilitators in the decision-making process [18,19,20]. Barriers are defined as “factors that hinder, limit, or prevent people from engaging in a certain behaviour”, whereas facilitators are “factors that favour, facilitate, or help people to engage in a certain behaviour” [21].

In the early oestrogen receptor (ER)-positive/human epidermal growth factor receptor 2 (HER2)-negative setting, long-term outcomes are overall favourable; nonetheless, a non-negligible proportion of patients experience recurrence, highlighting the need for therapeutic innovation to reduce recurrences and improve long-term survival. Ongoing phase III trials are testing oral selective ER downregulators in the context of extended adjuvant strategies, both upfront (e.g., CAMBRIA2, NCT05952557) and after 2–5 years of standard endocrine therapy (ET) (e.g., CAMBRIA1, NCT05514054, and EMBER4, NCT05774951) [22]. These switching designs in patients already receiving standard ET without evidence of recurrence pose specific enrolment challenges, as patients are asked to transition from an effective, familiar, generally well-tolerated treatment to an investigational agent whose benefit remains to be demonstrated. Importantly, these are disease-free patients in a preventive context, where the perceived relevance of additional medical treatment may be lower, potentially reducing interest in participation. Uncertainty about incremental benefit, new toxicities, and additional monitoring burden may further mitigate willingness to participate. Thus, there is a clear need to specifically navigate patient-level barriers and facilitators in this setting.

Several studies have examined barriers and facilitators relevant to decision-making about clinical trial participation. Frequently reported barriers include patient mistrust in the provider or health system, a lack of a patient-centred approach, poor patient-provider relationships, limited knowledge of clinical trials, time commitments, fear, and family issues [20,23,24,25]. Although these investigations have provided important insights, the available data are limited by the lack of valid and reliable tools specifically designed to assess barriers and facilitators for clinical trial participation. Identifying and addressing these factors is essential to accelerate innovation and ensure equitable access. Therefore, we aimed to develop and initially validate a questionnaire to assess barriers and facilitators among ER-positive/HER2-negative BC patients offered participation in CAMBRIA1 or EMBER4 trials.

## 2. Materials and Methods

**Figure 1:**
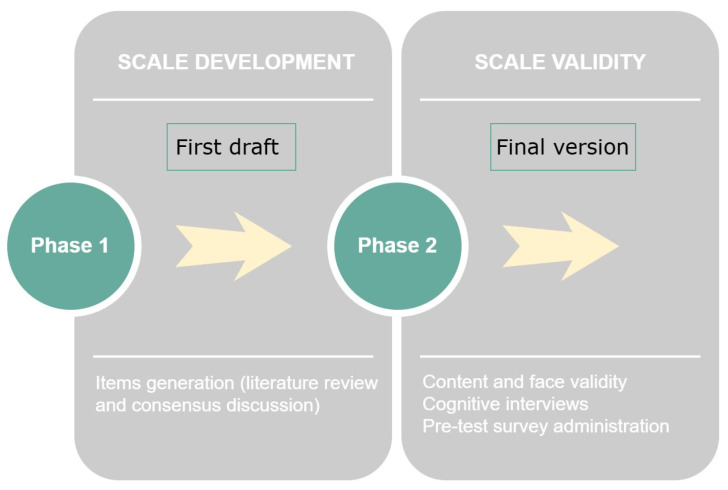
Study flow.

This study followed the methodology described by Rattray et al. [26], Tsang et al. [27], and Streiner et al. [28], and the best practice suggested by Boateng et al. [29] to develop and validate a new questionnaire assessing barriers and facilitators to clinical trial participation in individuals with breast cancer. As a first step, we identified the construct of interest, which, in the literature, is defined as an abstract representation of unobservable behaviour [30]. Once the construct was identified, we conducted an extensive search to identify existing questionnaires on the subject matter that fit the purpose. This step includes determining whether the instrument has been validated for the target population for which it was designed and whether the questionnaires adequately represent the constructs under examination. As the literature search revealed no existing questionnaires to address our specific setting, we undertook a validation study following the following phases from October 2024 to July 2025: (1) Instrument design and scale development, (2) Preliminary analyses of scale validity (Figure 1).

### 2.1. Phase 1: Instrument Design and Scale Development

#### 2.1.1. The Construct of Interest and the Conceptual Framework Definition

The construct under investigation was barriers and facilitators to clinical trial participation. Specifically, we intended to investigate factors influencing women’s clinical decision-making when they are already on effective BC treatment and are invited to participate in a clinical trial as an alternative treatment.

The Health Belief Model (HBM) [31,32] is a conceptual framework that explains patients’ participation behaviour in clinical trials. The HBM hypothesises that a health-related action depends mainly upon the value placed by a subject on a particular goal (value), the individual’s estimation of the likelihood that a certain activity would achieve that goal (expectancy), the desire and hope to get well (avoid sickness), the belief that a specific health action will restore health, prevent or ameliorate illness, and the individuals’ perception and motivation about health information by others (i.e., physicians). Therefore, the decision to participate in a clinical trial may be explained by the extent to which a patient perceives a threat to their health and believes that participating in the trial will effectively reduce that threat [31,32]. The framework summarises all these variables into the unique category of “Individual background and social context variables”, which includes general health perceptions and medical history, general evaluations and attitudes, and health locus of control, such as expectations towards the clinical trial. All these factors influence patients’ decisions to participate in clinical trials [32].

#### 2.1.2. Items Generation

The items were developed based on a framework derived from a recent overview of reviews that aimed to summarise barriers and facilitators to participation in clinical trials [20]. This review evaluated 753 primary studies, from which 881 barriers and facilitators emerged; these factors were then grouped into 20 main themes. Two qualitative studies investigating barriers and facilitators for clinical trial participation were also considered to identify additional themes [23,24]. These studies fit our purpose and allowed us to gain a comprehensive overview of potential factors and to consider patients’ perspectives in the instrument development. Based on these, a list of potential factors affecting patients’ participation in clinical trials was generated and adapted for our specific population.

The factors were discussed in a consensus meeting with five experts, who provided their opinions on the identified factors from the literature and on additional potential factors that could influence the decision to participate in a clinical trial in the specific context under investigation. Participants in the consensus discussion were selected through purposive sampling based on experience and knowledge in research and oncology: one methodologist, one study coordinator, one oncologist, one clinical trial nurse, and one oncology nurse. The consensus discussion was registered, and a verbatim transcription was undertaken to ensure the rigour and accuracy of the data [33,34]. A separate discussion was held with two cancer patients—not involved in the current trial but who had enrolled in other trials—to explore their perspectives on factors influencing their decision to participate in a clinical trial. The target population’s involvement in this phase could increase the chance that the items and other elements are representative of and relevant to the facets of the construct [35]. In this regard, using both inductive and deductive methods to define the domains and identify the questions is considered a best practice in scale development and validation [29]. All the experts confirmed the factors identified in the literature review and suggested one additional factor (shared decision-making), contextualising the literature findings for our study context and purpose. During the consensus discussion, these factors were then allocated into distinct categories, also based on literature findings: symptomatologic factors (side effects), contextual factors (trust in referral hospital), individual factors (previous experiences), clinical trial-related factors (study procedures), and relational factors with the clinician (trust).

From the literature review and consensus discussion, the factors were presented as both barriers and facilitators; therefore, some were barriers (e.g., “fear of change”) and others facilitators (e.g., “desire to heal”). We worded the items by defining the factors objectively and unbiasedly, thereby avoiding directing the patient a priori toward the concepts of barrier and facilitator. The definition by Rodríguez-Torres et al. was used to operationalise the concept of barriers and facilitators in formulating response options [20,36].

### 2.2. Phase 2: Preliminary Analysis of Scale Validity

#### 2.2.1. Content and Face Validity

The items underwent a content (quantitative) and face (qualitative) validity process [29]. Content validity refers to a measure’s ability to accurately assess the domain of interest [37]. Face validity refers to the extent to which respondents perceive the components of an assessment instrument as suitable for the intended concept and assessment aims [29]. For the content validity, the online Delphi technique was employed to achieve expert consensus [38,39,40]. This method involves evaluating agreement amongst rates concerning the relevance and appropriateness of the questionnaire’s items. This step involved an external panel of 10 experts (professional experts and target population) [29,35] to avoid bias in the assessment of items [41]: one methodologist (expert in scale validation), one clinical trial nurse, one oncology nurse, one psychologist, four oncologists, and two patients with cancer diagnosis. The experts were selected based on their expertise to ascertain the face and content validity of the initial version of the instrument, which was designed during the previous phases. Panel members were required to have specific training in oncology care and a minimum of 5 years of experience working with cancer survivors. The two external patients were individuals with solid cancer who had previously experienced involvement in clinical trials. Invitations were sent via email, explaining the study’s aim and their expected contribution.

For each item in the questionnaire, panel members were asked to rate “relevance” on a 4-point scale (1 = not relevant, 2 = somewhat relevant, 3 = quite relevant, 4 = highly relevant) and essentiality on a 3-point scale (1 = essential, 2 = useful but not essential, 3 = not necessary). The Content Validity Ratio (CVR) was calculated based on the three-point scale about essentiality; CVR values range from perfect disagreement (CVR = −1) to perfect agreement among panellists (CVR = +1). According to the current standards, the critical CVR threshold finds the lowest CVR value with a degree of agreement greater than 50% [42]. A cut-off value of 0.60 indicated the CVR threshold, based on the discrete binomial computation, while evaluating answers from 10 panellists with a type I error probability of 0.05 (using a one-tailed test) [42]. The content validity index for each item (I-CVI) and the scale (S-CVI) was calculated using a four-point relevance scale [43]. To obtain I-CVIs, we calculated the proportion of panellists who rated each item as completely relevant, divided by the total number of panellists; the S-CVI was calculated as the mean of all I-CVIs. I-CVIs and S-CVI equal to or higher than 0.70 were considered adequate [43].

#### 2.2.2. Interviews with Participants: Pre-Testing Questions

Cognitive interviews assessed respondents’ comprehension and answers to the drafted survey questions, identifying potential issues and insights [29,44]. This strategy ensures that responders understand the author’s intended information and gather it. This technique involved the oncologist and a subset of study participants (*n* = 5) to be consistent with the characteristics of the survey sample of interest [44,45]. The oncologist discussed the questionnaire’s items with the selected patients, utilising a verbal probing approach to assess comprehension, decision-making, memory retrieval, and response selection.

#### 2.2.3. Survey Administration: Pre-Testing on 56 Respondents

The questionnaire was pre-tested for reliability on 56 individuals in July 2025. The participants were Italian females with ER-positive/HER2-negative early BC who were offered the option to switch to the CAMBRIA-1 or EMBER-4 trials at Humanitas Research Hospital (NCT05774951 and NCT05514054) [22]. Of the 56 individuals enrolled in the pre-testing, 32 (57%) decided to participate in the trial, and 24 decided not to participate in the switch trials. A pre-test was essential to ensure that respondents interpreted the questions correctly and that the question order did not influence their answers, thereby reducing measurement error [46]. This step was also essential for estimating the time required to complete the questionnaire (response latency) [47]. For pre-testing, a sample of 12–50 respondents is recommended [48]. Participants received a brief introduction, highlighting the study’s aim, and were asked to provide overall feedback on the comprehensiveness of the survey’s items at the end. The participants were contacted via email, which contained an explanation of the purpose and the link to the questionnaire. The questionnaire administration procedures were the same as those hypothesised for the large-scale administration. The respondents matched the sociodemographic profile of the subsequent full-scale survey population, ensuring that the pre-test population was representative of it [49]. Cronbach’s alpha was calculated to assess the scale’s total reliability [50]. Items’ correlation was computed using the Pearson coefficient. The data were analysed using SPSS for Windows version 16.0 (SPSS, Chicago, IL, USA).

## 3. Results

### 3.1. Scale Validity

#### 3.1.1. Content and Face Validity

The panellists were eight healthcare professionals with at least 5 years of experience working with cancer survivors, and two oncology patients who had previously participated in at least 1 clinical trial. However, as patients tended to respond to the questions rather than provide judgments on content validity (despite a clear explanation of the investigation by the healthcare professional), we decided to consider their opinions only for face validity, to minimise bias in this phase. The healthcare panellists were mainly female (*n* = 7, mean age: 40 years); eight had postgraduate education, and two had doctoral degrees in research methodology and public health. All the healthcare panellists (except one) had a specific oncology education and were experts in cancer care. One of them was also a methodologist.

Two rounds of panel involvement were needed to obtain satisfactory CVRs, I-CVIs, and S-CVIs. After the first round of content validity, only 11 items (of 21) received CVR values ≥ 0.75; the remaining items obtained values ≤ 0.5. The critical CVR was 0.40. The I-CVIs were ≥0.75 in 16 items, whereas the I-CVIs were ≤0.625 in five items. The S-CVI was equal to 0.6. Based on these scores, the first draft of the questionnaire failed to achieve satisfactory indices of content validity. Therefore, the questionnaire structure was revised in light of comments on face validity. Some items were reformulated, and four were deleted. Revisions to the wording of the items were made to improve the questionnaire’s comprehensibility and readability. Considering the low scores, this phase required an additional consensus discussion to redesign the questionnaire. At the end of the second content validity round (Table 1), the critical CVR was ≥0.725 (despite four items receiving no satisfactory indexes), the I-CVIs were all higher than 0.70 (except item 9, which reached a score of 0.25), and the S-CVI was equal to 0.78. However, we decided not to remove item 9 (trust in a specific pharmaceutical industry of the experimental drug), as this item includes a factor listed as an influencing factor for clinical trial participation in Rodríguez-Torres’s overview of reviews [20].

#### 3.1.2. Participants’ Interview Results

After the participants responded to the content and face validity questionnaire, the oncologist discussed the items with the two patients who participated in the content and validity phase and three additional candidates for the survey administration. This step allowed us to refine and finalise the items’ structure regarding comprehensibility and item interpretation, thereby finalising the questionnaire structure. Respondents could provide answers that accurately reflect their point of view and the developers’ intended meaning. The participants responded positively to how the items were formulated and approved the final version of the questionnaire.

#### 3.1.3. Reliability of the Scale: Pre-Testing on 56 Respondents

Cronbach’s α was used to assess the questionnaire’s internal consistency in a pilot study with 56 respondents. The respondents were Italian females with a mean age of 52.4 years. Cronbach’s α was 0.874, indicating good internal consistency. There were no substantial differences in Cronbach’s α values when specific items were deleted. The item-total correlations ranged from 0.30 to 0.80, indicating that no items deviated from the standard and that each item showed an acceptable relationship with the total score [51]. The scale’s coefficient alpha value and item-total score correlations are given in Table 2.

### 3.2. Scale Description

#### 3.2.1. First Draft of the Questionnaire

Phase 1 of the scale development process produced the first draft of the questionnaire, comprising 21 items. Each item refers to a specific potential influencing factor. The response options comprised the following statements: “It facilitated me”, “it hampered me”, and “neutral” (no impact on the decision). These response options allowed us to determine if the mentioned factor was a facilitator (“It greatly facilitated me”, “it facilitated me”), a barrier (“it hampered me”, “it greatly hampered me”), or neither a barrier nor a facilitator (i.e., the factor did not influence the decision making). Additionally, the respondents had to indicate the extent to which the factor affected their decision on a scale from 1 to 5 (1 = little impact, 5 = fundamental impact), regardless of whether it was a barrier or a facilitator.

#### 3.2.2. Final Version of the Questionnaire

Phase 2 of the scale development process allowed us to define the final version of the questionnaire, which included 17 items (four items were removed because they were redundant). The questionnaire’s changes focused on the definitions and allocations of items to factors, as well as the response options and the factors’ grading system; these remained unchanged from the first draft. The wording of the items was widely revised, primarily based on the psycho-oncologist’s feedback. The questionnaire’s estimated completion time was about 20 min.

## 4. Discussion

This study presents an instrument for assessing barriers and facilitators to clinical trial participation among Italian BC patients. Barriers and facilitators are essential components of the decision-making process, representing a key aspect of oncology care, where a wide range of treatment options is offered to patients. This scale offers a window into the complex landscape of patient decision-making in oncology trials, revealing how individual priorities, clinical context, and broader life circumstances converge when women are invited to consider a switch in adjuvant ET. Systematically mapping these factors provides researchers and clinicians with actionable insights to anticipate patient concerns, tailor communication, and design patient-centred trials. Beyond recruitment metrics, the tool highlights the human dimension of trial participation, emphasising that informed, supported decision-making is essential for study success, empowering patients, and integrating innovative therapies effectively into everyday care. This study followed all necessary preliminary steps for the questionnaire’s initial validation, ensuring high methodological quality of the instrument from item development to reliability assessment [52].

A questionnaire’s content validity is evaluated during the initial stages of instrument development, including domain identification, item generation, instrument formation, and content validity quantification [38]. Validity is a critical factor in the selection and application of an instrument, as it is the degree to which evidence and theory support the interpretation of the results; in other words, it is the extent to which the instrument measures what it is designed to measure [53,54]. In our study, we adopted a three-stage approach, as it was recommended: (1) items’ development, (2) judgment quantification, (3) revising and reconstruction/reformation [38].

Research studies (mainly qualitative) on barriers and facilitators have described several potential influencing factors of the decision-making process in both oncology and non-oncology fields [19,20,23,24,25,55,56]. These studies allowed us to establish a solid basis for maximising the list of potential influencing factors during the consensus discussion to develop an evidence-based instrument. Although this evidence-based approach may appear simplistic and produce a distortion from reality, failing to consider additional potential factors and ignoring the interdependence of the various factors operating within complex social systems [57], it could represent a valid approach to rapidly map and assess potential influencing factors of the decision-making process since data are easy to collect, code, and analyse [58]. Efficiency is essential in survey methodology, where researchers attempt to obtain the attitudes of a representative sample for generalisation to a wider population [59]. However, in addition to closed questions, it is possible to include an ‘open’ question that invites respondents to provide additional information in free-text format [59].

After the first questionnaire draft was generated, we estimated its content validity, which is considered the most important measurement property because it quantifies whether the items are relevant, appropriate, and comprehensible to the construct of interest and the study population [60]. The content validity indexes were good, indicating that the items were relevant and representative of the measured construct. Different proposed techniques for quantifying content validity exist, such as adopting content validity-related indices and general agreement estimates [38]. Here, we adopted content validity-related indices, such as CVR, I-CVI, and S-CVI, which are among the most widely utilised and offer several advantages over other estimates [38]. The application of these measures raised concerns, as the original methods for calculating the CVR were not reported [42,61,62]. However, these indices are easy to compute, understand, and interpret, providing content validity for each item and the instrument, and allowing decisions on whether to retain or exclude items [38]. In our analysis, although some items were considered “not important” and “not appropriate” after the second round (behaviour of the clinician, trust in the pharmaceutical industry, religious beliefs, knowledge in the medical field), we decided to retain them in light of our previous considerations on the representativeness of all potential influencing factors and the questionnaire’s aim. However, based on the experts’ comments, the wording of these items was revised. At this stage, the adopted approach (revising and reconstruction/reformation) became essential for re-analysing the data and deciding whether to retain or omit specific items, regardless of the quantitative assessment’s content.

The questionnaire showed good test reliability. Reliability indicates how consistently a questionnaire produces the same results when used under similar conditions and over time [63]. Since the same instrument, used in a different setting or with different subjects, can demonstrate wide variation in reliability [64], we ensured that the pre-test population of this investigation was representative of the future full-scale survey population. This property can be estimated in various modalities [53,63,65]. We used Cronbach’s alpha to assess internal consistency and to calculate correlations among questionnaire responses [66]. This coefficient is usually applied to written tests where the internal test consistency is the most important property [65]. High item-total correlations indicated that the items measure the same construct as the overall scale, supporting the instrument’s overall reliability. By analysing these statistics, researchers can identify items that may need revision to improve the overall quality and validity of the measurements [67]. Our analyses indicated that items 7 (randomisation process, r = 0.179) and 13 (religious beliefs, r = 0.088) were far below the accepted threshold of r = 0.30, suggesting the need to delete these items in future validation works [7].

This study presents several limitations. Firstly, the small sample size limited the accuracy and generalizability of the results and prevented further evaluation of the instrument’s psychometric properties (exploratory factor analysis). This questionnaire was designed to understand the barriers and facilitators of recruitment to a specific RCT; therefore, the number of individuals involved was equal to those enrolled in this trial. Nevertheless, the sample size was appropriate for administering a pre-testing survey to gather preliminary validity indices before determining whether to conduct a future large-scale study utilising this instrument. Secondly, the questionnaire’s structure was based on a predefined list of potential influencing factors from the literature, which may have prevented the examination of additional factors. However, these factors were discussed in a consensus session, and an open question was added at the end of the questionnaire to identify additional relevant factors to be included. Thirdly, the content validity assessment included two patients on the panel who represented the same target population as the planned survey administration. This choice may yield biassed results, as patients’ personal involvement may lead them to respond to the questions rather than judge the items’ relevance and appropriateness. However, these patients were trained about the objective of this validation phase, and after completing the questionnaire, their responses were rechecked by telephone to ensure they answered the questions correctly. Fourth, adopting a 3-point Likert scale for the first and second rounds of the content validity phase, rather than a 4- or 5-point scale, may be debatable, depending on the references and authors’ views [38]. However, a 5- or 3-point rating scale is recommended (especially in the first round) to prevent ambivalent or forced ratings when no “neutral” option is available [68]. Using a 4-point Likert scale [69] implies collapsing four ordinal levels into two dichotomous categories (ratings 1 and 2: content is “invalid”; ratings 3 and 4: content is “valid”) and augments the likelihood that the experts will agree by chance alone 50% of the time, irrespective of the number of experts [38]. Fifth, the cognitive interview subsample (*n* = 5) was small, reflecting the minimum required for conducting cognitive interviews in a scale development [7]. Furthermore, the cognitive interview transcripts were not verbatim. Although the oncologist took notes on the key concepts, we might have overlooked some pertinent information. However, the qualitative data were not the primary focus of this research and could be further explored in future ad hoc investigations. Sith, a 20 min completion time is non-trivial for patients on active treatment. A brief participant feedback on acceptability could have strengthened the pre-testing section. However, this aspect will be considered for future research validation steps. Finally, Item 9 (Trust in the pharmaceutical industry) was retained despite receiving an I-CVI of 0.25, which is significantly lower than the 0.70 threshold, due to its presence in the literature. Although a citation does not easily override expert near-consensus against an item’s relevance, the literature supporting this theme was a solid overview of reviews that included 753 primary studies. To effectively understand whether this aspect can be an influencing factor, it is currently essential to retain it for future large-scale investigations.

## 5. Conclusions

We developed a questionnaire to assess barriers and facilitators to clinical trial participation among women with ER-positive/HER2-negative early BC, in the context of adjuvant ET switch trials. A greater understanding of such determinants may inform trial design and enrolment, as well as drug development in BC management, and support the tailoring of patient engagement strategies to facilitate informed participation and reduce the risk of premature trial termination due to poor accrual in an era of substantial financial investment in oncological research. Our study provides evidence of the scale’s content validity and internal consistency by following a comprehensive, multiphase methodology that includes expert consensus and an initial psychometric evaluation. Beyond its immediate application, this instrument provides a practical framework for understanding patient decision-making, informing both trial design and strategies to enhance enrolment while respecting patient autonomy. Since preliminary internal consistency has been established, future research will involve an exploratory factor analysis in a larger cohort to examine the underlying factor structure, further establish construct validity, and enable large-scale psychometric evaluations, ultimately supporting more patient-centred approaches in oncology research.

## Figures and Tables

**Table 1:** Content validity indexes.

Experts (*n* = 8)		CVR				CVI			
Item Number	Items	Ne	Critical CVR	Observed CVR	Interpretation	Ratings ≥ 3	I-CVIs	S-CVI	Interpretation
Item 1	Side effects of therapy	8		1	Important	8	1		Appropriate
Item 2	Prospective of having a new treatment option	8		1	Important	8	1		Appropriate
Item 3	Received information	8		1	Important	8	1		Appropriate
Item 4	Behaviour of the clinician	6		0.5	Not important	7	0.875		Appropriate
Item 5	Sharing the decision-making process with the clinician	8		1	Important	8	1		Appropriate
Item 6	Trust in the referral hospital	8		1	Important	8	1		Appropriate
Item 7	Randomisation process	8		1	Important	8	1		Appropriate
Item 8	Trust in the research	8		1	Important	8	1		Appropriate
Item 9	Trust in the pharmaceutical industry	4	0.725	0	Not important	2	0.25	0.78	Non-appropriate
Item 10	Access to innovative treatments	8		1	Important	8	1		Appropriate
Item 11	Clinical monitoring	8		1	Important	8	1		Appropriate
Item 12	Commitment to rigorous timepoints	8		1	Important	8	1		Appropriate
Item 13	Religious believe	6		0.5	Not important	6	0.75		Appropriate
Item 14	Previous personal experiences in the treatment journey	8		1	Important	8	1		Appropriate
Item 15	Support from parents or friends	8		1	Important	8	1		Appropriate
Item 16	Peer-to-peer support	8		1	Important	8	1		Appropriate
Item 17	Knowledge in the medical field	6		0,5	Not important	6	0.75		Appropriate

Cut-off value of 0.60 represents the CVR threshold; I-CVI and S-CVIs ≥ 0.70 are considered appropriate.

**Table 2:** Items correlation and scale reliability.

	Items	M	SD	Total Item Correlation	R^2^	Scale Coefficient of Reliability If the Item Is Deleted
Item1	Side effects of therapy	1.91	0.880	0.517	0.507	0.866
Item 2	Prospective of having a new treatment option	1.71	0.847	0.417	0.417	0.870
Item 3	Received information	1.54	0.873	0.678	0.656	0.860
Item 4	Behaviour of the clinician	1.71	0.948	0.716	0.761	0.857
Item 5	Sharing the decision-making process with the clinician	1.73	0.944	0.654	0.738	0.860
Item 6	Trust in the referral hospital	1.63	0.926	0.696	0.654	0.859
Item 7	Randomisation process	2.39	0.846	0.179	0.266	0.879
Item 8	Trust in the research	1.82	0.917	0.667	0.637	0.860
Item 9	Trust in the pharmaceutical industry	2.66	0.745	0.439	0.392	0.869
Item 10	Access to innovative treatments	1.70	0.913	0.739	0.857	0.857
Item 11	Clinical monitoring	1.57	0.850	0.697	0.760	0.859
Item 12	Commitment to rigorous timepoints	1.89	0.846	0.404	0.400	0.871
Item 13	Religious belief	2.89	0.454	0.088	0.215	0.878
Item 14	Previous personal experiences in the treatment journey	1.88	0.974	0.481	0.360	0.868
Item 15	Support from parents or friends	2.02	1.000	0.302	0.376	0.876
Item 16	Peer-to-peer support	2.54	0.852	0.332	0.336	0.874
Item 17	Knowledge in the medical field	2.41	0.890	0.416	0.323	0.871
	Total items			Cronbach α = 0.874		

Abbreviations: M = Mean, SD = standard deviation.

## Data Availability

The data that support the findings of this study are not openly available due to reasons of sensitivity and are available from the corresponding author upon reasonable request.

## Abbreviations

The following abbreviations are used in this manuscript:

ET	Endocrine Therapy
BC	Breast Cancer
ER	Oestrogen Receptor
HBM	Health Belief Model
CVR	Content Validity Ratio
CVI	Content Validity Index
